# Molecular imaging of cardiac CXCR4 expression in a mouse model of acute myocardial infarction using a novel ^68^Ga-mCXCL12 PET tracer

**DOI:** 10.1007/s12350-020-02262-6

**Published:** 2020-07-16

**Authors:** Mathias Johannes Zacherl, Andrei Todica, Carmen Wängler, Ralf Schirrmacher, Mohammad Ali Hajebrahimi, Joachim Pircher, Xiang Li, Simon Lindner, Matthias Brendel, Peter Bartenstein, Steffen Massberg, Stefan Brunner, Sebastian Lehner, Marcus Hacker, Bruno C. Huber

**Affiliations:** 1 Department of Nuclear Medicine, University Hospital of Munich, LMU Munich, Munich, Germany; 2 Biomedical Chemistry, Department of Clinical Radiology and Nuclear Medicine, Medical Faculty Mannheim of Heidelberg University, Mannheim, Germany; 3 Department of Oncology, Division of Oncological Imaging, University of Alberta, Edmonton, AB Canada; 4 Department of Cardiology, University Hospital of Munich, LMU Munich, Munich, Germany; 5 German Centre for Cardiovascular Research (DZHK), Partner Site Munich Heart Alliance, Munich, Germany; 6 Department of Biomedical Imaging and Image-guided Therapy, Division of Nuclear Medicine, Medical University of Vienna, Währinger Gürtel 18-20, 1090 Vienna, Austria; 7 Ambulatory Healthcare Center Dr. Neumaier & Colleagues, Radiology, Nuclear Medicine, Radiation Therapy, Regensburg, Germany

**Keywords:** Myocardial infarction, CXCR4/CXCL12 chemokine axis, mice, PET, tracer, ^68^Ga

## Abstract

**Background:**

The chemokine receptor CXCR4 and its ligand CXCL12 have been shown to be a possible imaging and therapeutic target after myocardial infarction (MI). The murine-based and mouse-specific ^68^Ga-mCXCL12 PET tracer could be suitable for serial in vivo quantification of cardiac CXCR4 expression in a murine model of MI.

**Methods and Results:**

At days 1-6 after MI, mice were intravenously injected with ^68^Ga-mCXCL12. Autoradiography was performed and the infarct-to-remote ratio (*I*/*R*) was determined. In vivo PET imaging with ^68^Ga-mCXCL12 was conducted on days 1-6 after MI and the percentage of the injected dose (%ID/g) of the tracer uptake in the infarct area was calculated. ^18^F-FDG-PET was performed for anatomical landmarking. Ex vivo autoradiography identified CXCR4 upregulation in the infarct region with an increasing *I*/*R* after 12 hours (1.4 ± 0.3), showing a significant increase until day 2 (4.5 ± 0.6), followed by a plateau phase (day 4) and decrease after 10 days (1.3 ± 1.0). In vivo PET imaging identified similar CXCR4 upregulation in the infarct region which peaked around day 3 post MI (9.7 ± 5.0 %ID/g) and then subsequently decreased by day 6 (2.8 ± 1.0 %ID/g).

**Conclusion:**

Noninvasive molecular imaging of cardiac CXCR4 expression using a novel, murine-based, and specific ^68^Ga-mCXCL12 tracer is feasible both ex vivo and in vivo.

**Electronic supplementary material:**

The online version of this article (10.1007/s12350-020-02262-6) contains supplementary material, which is available to authorized users.

## Introduction

In recent years, there has been a growing interest in a better understanding of the inflammatory processes after myocardial infarction (MI) through employing molecular imaging techniques.^1^,^2^ Several studies have used the well-established and broadly available PET tracer 2-deoxy-2-[^18^F]fluoro-d-glucose (^18^F-FDG) and these studies were able to show enhanced metabolic activity in inflammatory cells.^3^ However, a major limitation of ^18^F-FDG PET is the non-specific uptake in cells with glycolytic metabolism and the requirement of protocols to suppress physiological myocardial glucose uptake.^4^

More recently, the CX-motive chemokine receptor type 4 (CXCR4) has been introduced as suitable target for molecular imaging, which possibly allows to directly assess the extent of inflammatory activity.^5^ CXCR4 is a member of the G-protein-coupled receptor family and plays a pivotal role in hematopoiesis, organogenesis, and vascularization during development^6^ while it directs cells toward higher concentrations of chemokines.^7^,^8^ Dysregulation of the chemokine receptor CXCR4 and its ligand CXCL12, also known as stromal cell-derived factor 1 (SDF-1), leads to the development of many human diseases, including cancer, immunodeficiency, and autoimmune and chronic inflammatory diseases.^9^-^11^ CXCR4 is strongly expressed by leukocytes, including granulocytes, monocytes, T cells, B cells, and natural killer cells as well as by bone-marrow-derived progenitor cells.^12^

The CXCL12/CXCR4 axis has been shown to play a pivotal role during cardiovascular development, cardiac repair, and tissue homeostasis after ischemia.^12^ However, it seems that the CXCL12/CXCR4 axis has a more complex and double-edged role in cardiovascular disease. On one hand, previous and current data demonstrate that activation of CXCL12/CXCR4 signaling leads to attenuation of ischemic cardiomyopathy by tissue protective effects, increased neovascularization, reduced infarct size, and an improved heart function after MI.^12^,^13^ On the other hand, the CXCL12/CXCR4 axis has also been demonstrated to have a negative impact on cardiac remodeling after myocardial infarction, potentially associated with the recruitment of pro-inflammatory cells to the ischemic region.^14^ Also, two previous studies investigating a blockade of CXCR4 with the small molecule antagonist AMD3100 generated conflicting results. A continuous blockade of CXCR4 with AMD3100 resulted in impaired survival and reduced cardiac function after acute MI^15^,^16^, whereas a single-time treatment caused improved healing and functional recovery.^16^,^17^

Hence, the exact role of the chemokine receptor CXCR4 in tissue repair is still undefined and therefore noninvasive imaging of CXCR4 expression after acute MI is of substantial value. For this purpose, we applied a novel, mouse-specific ^68^Ga-mCXCL12 tracer to identify myocardial CXCR4 expression in a mouse model of acute MI.

## Materials and Methods

### Tracer Synthesis

**Figure 1:**
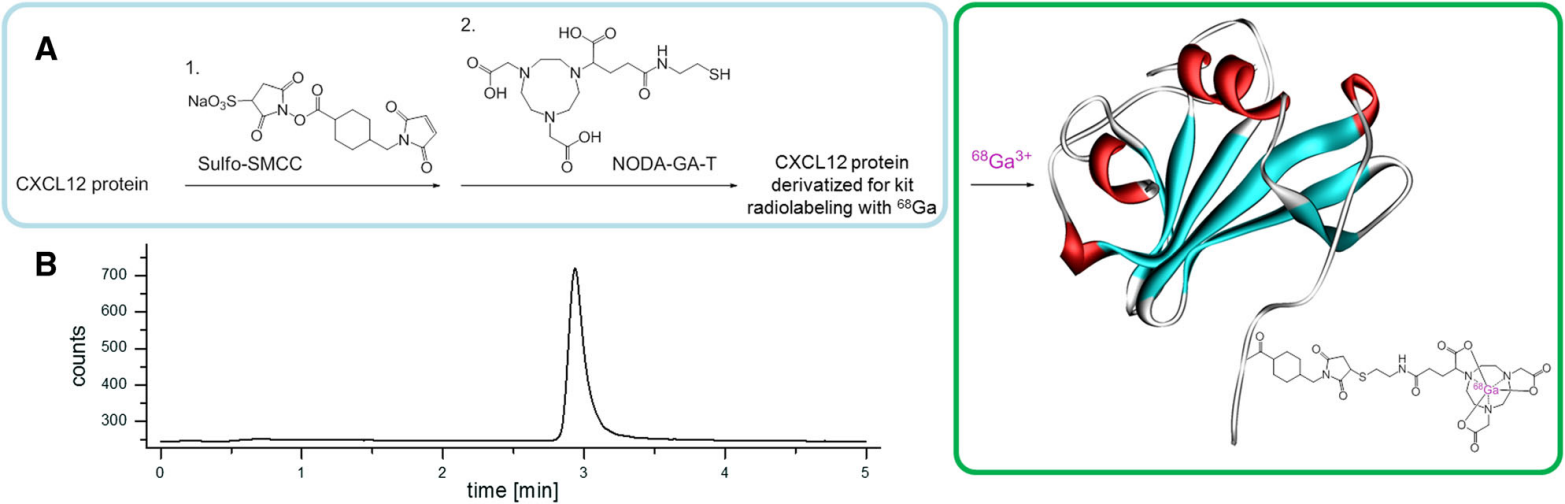
Schematic depiction of tracer synthesis. (**A**) Preparation of the NODA-GA-T-modified mCXCL12 protein (PDB ID: 1A15^33^) labeling precursor (blue) and the ^68^Ga-kit-radiolabeling step (green). (**B**) Analytical radio-HPLC chromatogram of ^68^Ga-mCXCL12

Recombinant murine CXCL12 (mCXCL12, PeproTech Germany, Hamburg, Germany) was kit-like labeled as previously described.^18^ In brief, amino functionalities of the protein were randomly first derivatized with sulfo-SMCC, a maleimide-bearing crosslinking agent and afterwards reacted with NODAGA-thiol. After purification of the protein, it can be stored until the labeling is performed. By introducing only 1.6 derivatization sites per molecule, the binding characteristic of the derivatized mCXCL12 should remain preserved.^18^ For the radiolabeling reaction, 240 to 340 MBq ^68^Ga were obtained by fractional elution of a commercially available ^68^Ge/^68^Ga generator (Eckert & Ziegler, Berlin, Germany) in 1 mL of 0.1 M HCl. The pH of the solution was adjusted to 3.5 to 4.0 by adding approximately 85 mL of a 1.25 M sodium acetate solution. Subsequently, a solution of the NODAGA-T-derivatized protein (6.9 to 10 nmol) in HEPES buffer (0.025 M, pH 4.0) was added and incubated for 7 min at room temperature. After radiolabeling, HEPES buffer (2 M;150 mL) was added to this mixture to adjust the pH to 7.0, and the solution was filtered sterile. The radiolabeled protein was analyzed by analytic radio-HPLC (gradient of 0% to 100% MeCN + 0.1% TFA in 5 min). A schematic depiction of the preparation of the precursor and the kit-like labeling procedure is shown in Fig. 1.

### Animal Model

Myocardial infarction was induced in male C57BL/6 mice (Charles River Laboratories, Sulzbach, Germany) at 8 to 12 weeks of age, by permanently ligating the proximal left anterior descending artery (LAD) as previously described.^19^ In short, mice were anesthetized by intraperitoneal (i.p.) injection of a mixture of 100 mg/kg ketamine (Sigma Chemical Co., St. Louis, MO) and 5 mg/kg Xylazine (Sigma-Aldrich, Munich, Germany), intubated, and artificially ventilated by a mouse ventilator (HUGO SACHS, March, Germany) with 200 strokes/min. Animal care and all experimental procedures were performed in strict accordance to the German and National Institutes of Health animal legislation guidelines and were approved by the local animal care and use committees.

### In Vitro Autoradiography with ^68^Ga-mCXCL12

To investigate the specificity of the ^68^Ga-mCXCL12 binding, an in vitro autoradiography experiment was performed. Therefore, two days after LAD ligation, induction of anesthesia was performed as described previously and mice were sacrificed by cervical dislocation. The heart was excised, rinsed with cold physiologic saline solution, and then frozen in Tissue-Tek (Embedding Medium for Frozen Tissue Specimens, Sakura Finetek USA, Torrance, CA). Hearts (*n* = 4) were then cut using a cryostat (Leica CM1510, Nussloch, Germany) set at − 20 °C into serial short-axis sections (20 μm thickness each), which were thaw-mounted on glass slides. Slides were dried at room temperature for 60 minutes. For blocking ^68^Ga-mCXCL12 binding, 30 slides were incubated for 30 minutes with 0.15 mM CXCR4 antibody (hCXCR4-PE, R&D Systems, Minneapolis, Minnesota, USA). An additional 30 slides were incubated for the same amount of time with HEPES buffer solution and served as controls. Each slide was carefully washed with HEPES and then incubated with 0.5 mM ^68^Ga-mCXCL12 for 45 minutes.^20^ Slides were washed again three times with HEPES and then exposed to autoradiographic imaging plate (Fujifilm MS Imaging Plates in a Fujifilm BAS Cassette 2 2025, Fujifilm Europe, Düsseldorf, Germany) for at least 12 hours. The imaging plate was scanned at 25 μm resolution with Raytest equipment (CR 35 BIO, Dürr Medical, Germany), and analyzed with AIDA Image Analysis software V4.50 (Elysia-Raytest, Straubenhardt, Germany). Regions-of-interest (ROIs) were drawn within the infarcted and remote myocardium to calculate the infarct-to-remote ratios (*I*/*R*).

### Ex Vivo Autoradiography with ^68^Ga-mCXCL12

To visualize and analyze the time course of ^68^Ga-mCXCL12 accumulation, *ex vivo* autoradiography was performed in mice after 12 hours (*n* = 2), on day 1 (*n* = 6), day 2 (*n* = 4), day 3 (*n* = 5), day 4 (*n* = 4), day 5 (*n* = 4), and day 6 (*n* = 4) and day 10 (*n* = 3) after myocardial infarction. After induction of anesthesia, as mentioned above, 20 ± 2 MBq of ^68^Ga-mCXCL12 were injected into a tail vein. Mice were sacrificed by cervical dislocation at 45 minutes after tracer injection (also refer to supplement); the heart was excised, rinsed with cold physiologic saline solution, and then cut and prepared as described above. Within 30 minutes (i.e., 120 minutes after tracer injection), the slides were placed on an autoradiographic imaging plate (Fujifilm MS Imaging Plates in a Fujifilm BAS Cassette 2 2025, Fujifilm Europe, Düsseldorf, Germany) for at least 12 hours. Readout was performed and *I*/*R* were calculated as described above.

### In Vivo PET Imaging

**Figure 2:**
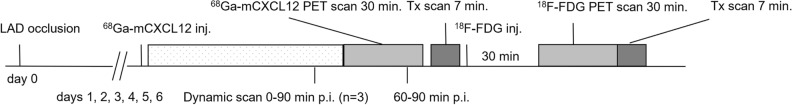
Experimental design of the in vivo PET Imaging. ^68^Ga-mCXCL12 PET was performed on day 1 to 6 over 30 min. 60 min. p.i. followed by a 7 min. transmission (Tx) scan, three mice were scanned dynamically over 90 min; subsequently an additional 30-min ^18^F-FDG-PET scan was acquired for landmarking

^68^Ga-mCXCL12 and ^18^F-FDG imaging was performed on a dedicated small-animal microPET scanner (Inveon Dedicated PET, Preclinical Solutions, Siemens Healthcare Molecular Imaging, Knoxville, TN, USA) on day 1 (*n* = 3), day 2 (*n* = 6), day 3 (*n* = 3), day 4 (*n* = 3), day 5 (*n* = 3), and day 6 (*n* = 3) after LAD artery occlusion. To avoid unnecessary stress for the rodents and keep drop-out rates low, no longitudinal measurements were performed. For scanning, anesthesia was induced with isoflurane (2.5%), and subsequently maintained with isoflurane (1.5%) delivered in pure oxygen at a rate of 1.2 L/min via a face mask without intubation. A volume of approximately 100 μL with 20 ± 3 MBq of ^68^Ga-mCXCL12 were injected into a tail vein after placing an intravenous catheter and flushed with 50 μL of saline solution. Animals were placed within the aperture of the PET scanner. The body temperature was monitored using a rectal thermometer and was held within the normal range using a heating pad. A three-dimensional list-mode acquisition was initiated lasting from minutes 60 to 90 after tracer injection followed by a seven-minute transmission scan performed with a rotating [^57^Co] source for attenuation and scatter correction. To estimate the optimal imaging time point with a high infarct-to-remote ratio in the heart, dynamic scans lasting from minutes 0 to 90 were acquired. For anatomical landmarking and to delineate the infarcted myocardium, an additional ^18^F-FDG PET scan, lasting 30 minutes, was subsequently initiated. Therefore, 20 MBq ^18^F-FDG were injected in a volume of 100 μL, as previously described^21^ (Fig. 2). Recovery from anesthesia and the PET scan was monitored in the home cage and overseen by a veterinarian.

All data were processed with the Inveon Acquisition Workplace (Siemens Medical Solutions, Knoxville, TN). The ^68^Ga-mCXCL12 data were iteratively reconstructed as static image using ordered-subsets expectation maximization (OSEM) 3D (four iterations) and MAP (32 iterations) image reconstruction algorithms. The final images consisted of a 256 × 256 matrix (159 slices, thickness 0.796 mm) with a zoom factor of 100% and a *β* of 0.15 as previously established by our group.^21^^18^F-FDG PET data were reconstructed as a static image using the same reconstruction algorithm as described above. ^68^Ga-mCXCL12 biodistribution data were reconstructed as framed images (3 × 10 min, 2 × 30 min) using the same algorithm. All data were normalized and corrected for random coincidences, dead time and decay, as well as attenuation.

### PET Image Analysis

The ^68^Ga-mCXCL12 PET scans were analyzed using the Inveon Research Workplace (Siemens Medical Solutions, Knoxville, TN). The biodistribution of ^68^Ga-mCXCL12 in the blood pool and the infarcted myocardium was evaluated (*n* = 3; day 1, 4, and 6) to estimate the optimal timeframe for further analysis, indicating an optimal infarct-to-remote ratio lasting from 60 to 90 minutes. Therefore, standard volumes of interest (VOI) were drawn in the last frame (60 to 90 minutes) in the infarcted area, using a co-registration with the ^18^F-FDG pet scan for landmarking. To avoid spill-over from the liver, the VOI was placed in the front wall of the heart, in the infarcted myocardium. The correct placement of the VOI was verified in axial, coronal, and sagittal projections. The mean radioactivity concentration was quantified as the percentage of the injected dose per gram of tissue (%ID/g). Another VOI was drawn within the remote, healthy myocardium in the *basal anterior* wall of the heart to avoid spill-over from the liver and the %ID/g was calculated likewise to serve as an internal control. Furthermore, time-activity curves were calculated for the infarcted area, the remote myocardium, the liver, the bladder, and the right limb to demonstrate the biodistribution of ^68^Ga-mCXCL12.

### Validation of Radiotracer Specificity by Blocking with Native mCXCL12

After synthesis of ^68^Ga-mCXCL12, a 10 times higher molar excess of native mCXCL12 was dissolved and co-injected with 15 MBq ^68^Ga-mCXCL12 on day 2 after myocardial infarction (*n* = 2). After 60 minutes, a 30-min PET scan was performed. A ^18^F-FDG scan was conducted subsequently as previously described. For autographic analysis, an additional mouse received the same co-injection with mCXCL12 and the heart was extracted after 45 min. Autoradiography was carried out as described above (Fig. 4C and D).

### Immunofluorescence Analysis

For immunofluorescence analysis, hearts were harvested 3 days after arterial ligation and were immediately embedded in OCT and frozen at − 80 °C. Frozen samples were cut with a cryotome (Leica CM1510, Nussloch, Germany) into 10 μm sections, fixed with 4% formaldehyde, and blocked with goat serum. The sections were incubated with monoclonal PE-conjugated anti mouse CXCR4 (clone2B11, eBioscience) or respective PE-labeled isotype control (Rat IgG2b kappa, eBioscience) for 1 hour at room temperature. DNA was stained with 1 µg/mL DAPI (Sigma), and a coverslip was placed using mounting medium (DAKO). Primary antibodies were applied 1:100 (final dilution). Images were acquired using a LSM 880 confocal microscope with Airyscan module and Plan-Apochromat 20×/0.8 air objective (Carl Zeiss Microscopy) and processed using ZEN software (Zeiss).

### Statistical Analysis

Group comparisons of *I*/*R* in autoradiography and %ID/g in PET results were performed using one-way ANOVA and the Holm-Bonferroni method was used to correct for multiple comparisons, calculated by IBM SPSS 25 Statistics. Shapiro-Wilk was used to test for normal distribution. A paired Student’s *t* test was used to compare data between healthy and infarcted myocardium in the ^68^Ga-mCXCL12 PET. In general, a threshold of *P* < 0.05 was considered to be significant for rejection of the null hypothesis.

## Results

### Tracer Synthesis, In Vivo Biodistribution, In Vitro Autoradiography, and Blocking with Native mCXCL12 Co-injection to Test Target Specificity

The protein mCXCL12 was prepared for labeling by introducing only 1.6 derivatization sites per molecule. Before every experiment, mCXCL12 was labeled using a kit-like labeling technique with ^68^Ga. Consequently, the radiotracer was analyzed by analytical radio-HPLC (gradient of 0% to 100% MeCN + 0.1% TFA in 5 min) and found to be 95% to 99% pure, the molar activity of the tracer was between 20 and 45 GBq/µmol. The molecular weight is 8.3 kDa and mCXCL12 consists of 68 amino acids.

**Figure 3:**
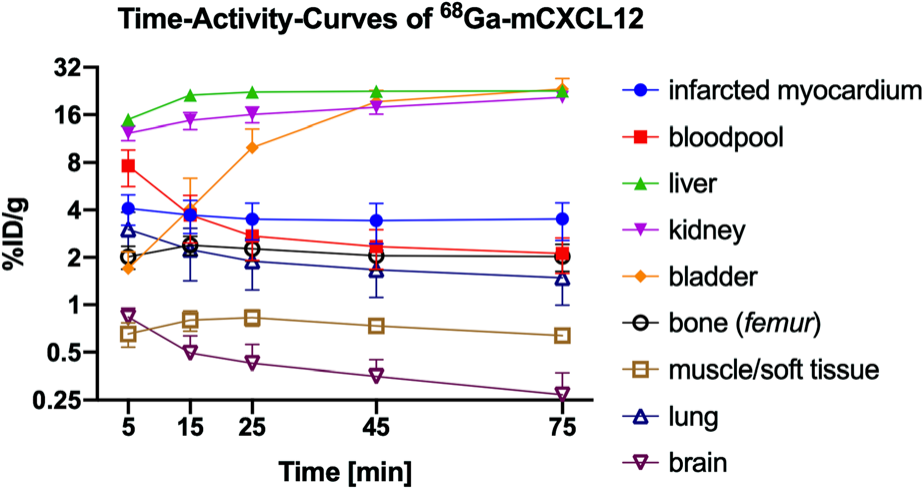
^68^Ga-mCXCL12 time-activity curves. ^68^Ga-mCXCL12 PET time-activity curves over 90 min (three 10-min, two 30-min frames) in the blood pool, the myocardium and several tissues of the mouse. All data represent mean ± SEM (*n* = 3)

Representative time-activity curves for ^68^Ga-mCXCL12 are presented in Fig. 3 with volumes of interest placed in the infarcted myocardium, blood pool (myocardial cavity), liver, kidney, bladder, bone (*femur*), muscle, lung, and brain. Radioactivity concentration in the liver consistently exceeded that of all other organs. Blood pool activity decreased constantly within the first 30 minutes and showed afterwards only a slight decrease until the end of the study. A relatively constant phase of radioactivity distribution was reached after approx. 45 minutes with minor changes up to 60 minutes after tracer injection.

**Figure 4:**
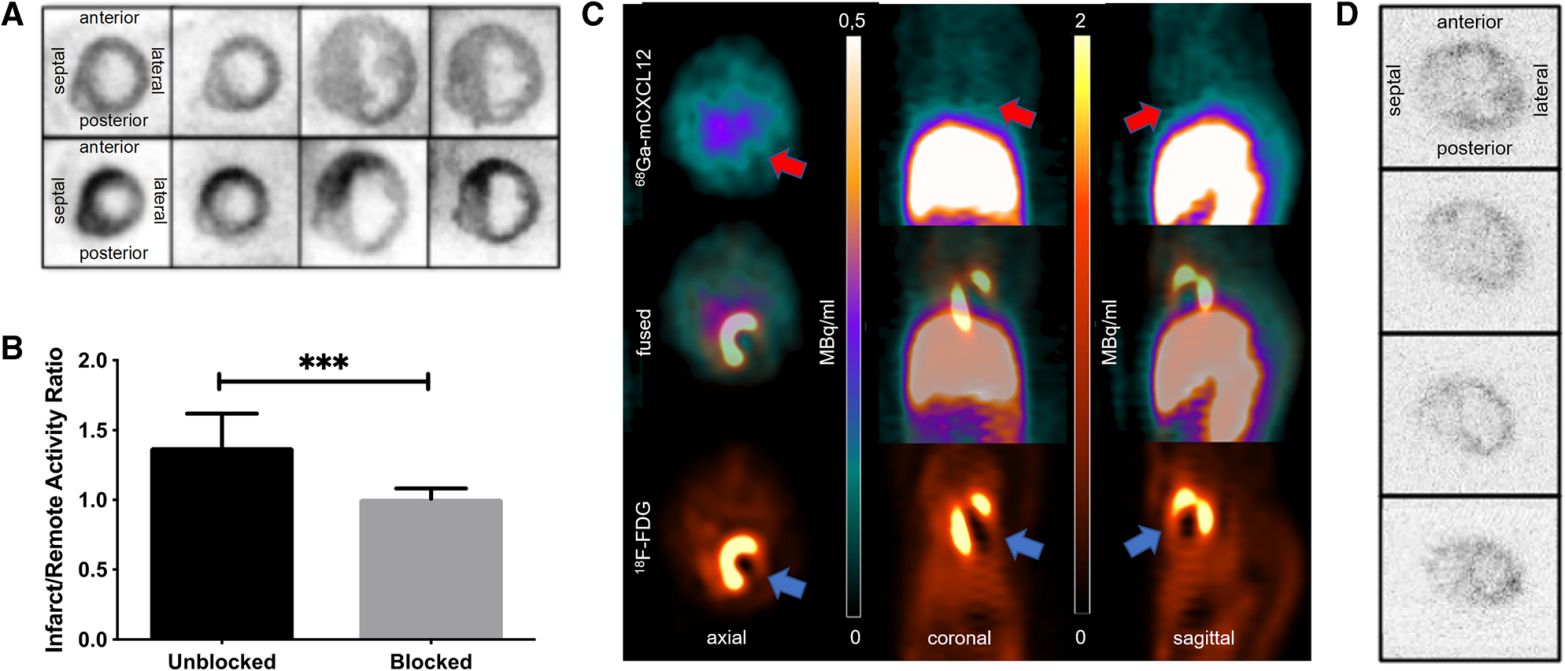
Specificity of ^68^Ga-mCXCL12. (**A**) Unblocked slices with ^68^Ga-mCXCL12 accumulation in the infarcted heart in the *anterior* and *antero-septal* wall (bottom row). After blocking with CXCR4 antibody sections show no visible ^68^Ga-mCXCL12 uptake anymore (top row). (**B**) Significantly higher ^68^Ga-mCXCL12 myocardial infarct-to-remote activity ratios as compared to the unblocked slices. All data represent mean ± SD. (**C**) PET image on day 2 shows no visible ^68^Ga-mCXCL12 uptake in the infarcted myocardium after Co-Injection with excess of native mCXCL12. ^18^F-FDG PET showing the viable myocardium (red arrow: no ^68^Ga-mCXCL12 uptake; blue arrow: infarcted myocardium). (**D**) Autoradiography on day 2 after Co-Injection with excess of native mCXCL12 shows no significant ^68^Ga-mCXCL12 uptake (top to bottom shows representative slides from the base to the apex of the heart)

Target specificity of the ^68^Ga-mCXCL12 tracer was evaluated using consecutive sections of the same heart of previously infarcted animals. Sections were either incubated with CXCR4 antibody or saline as control. Figure 4A (bottom row) demonstrates an increased tracer uptake in the *anterior* and *antero-septal* wall within the infarcted myocardium (LAD-territory). After blocking with CXCR4 antibody, comparative sections of the same heart area showed a fully suppressed ^68^Ga-mCXCL12 tracer uptake (Fig. 4A, top row). Semiquantitative assessment of the antibody-blocked slides revealed a significantly lower *I*/*R* of 1.01 ± 0.01 as compared to the unblocked control slides (1.38 ± 0.23; *P* < 0.001; Fig. 4B). To further address the specificity of our radiotracer, we performed competition studies with excess of native mCXCL12 for in vivo imaging and autoradiography. As can be seen in Fig. 4C and D, both experiments revealed complete blocking of radiotracer accumulation in the infarct region and thus confirmed the CXCR4-specific uptake of the newly developed ligand.

### Ex Vivo Molecular Imaging of Cardiac CXCR4 Expression by Autoradiography

**Figure 5:**
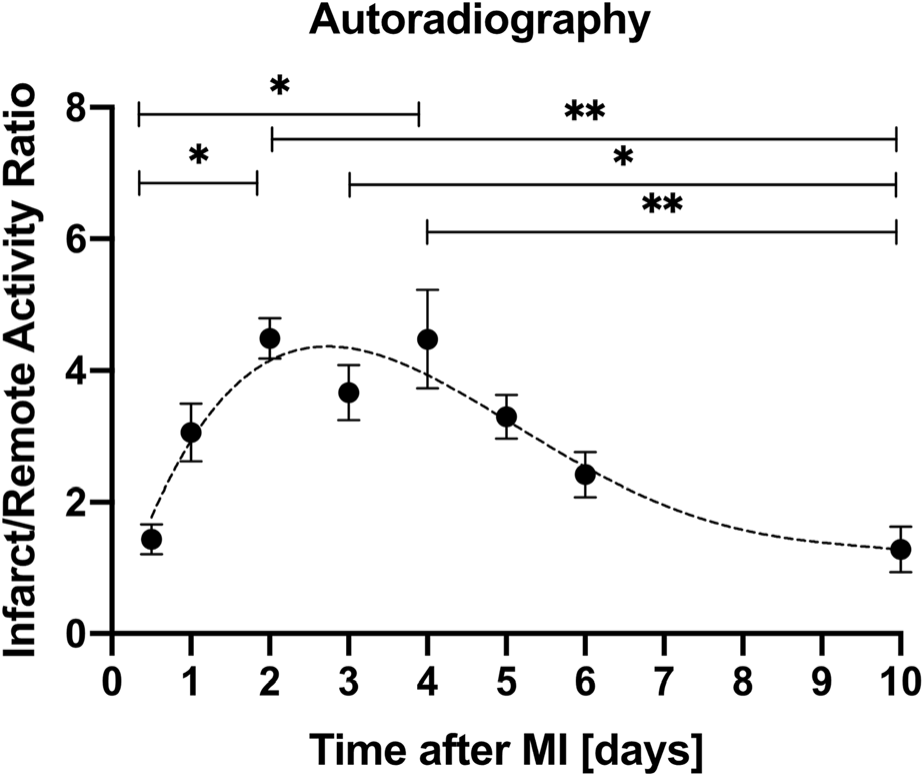
Time-course of CXCR4 expression in autoradiography. After a significant increase until day 2 CXCR4 infarct-to-remote activity ratios (*I*/*R*) significantly decrease from day 4 to day 10. For better visualization of the CXCR4 kinetic (*I*/*R*) over time a fourth-order polynomial regression trendline was calculated. All data represent mean ± SEM

To visualize and analyze the time course of cardiac ^68^Ga-mCXCL12 accumulation, one-way ANOVA was performed (methods), showing significant differences for *I*/*R* among time points (*P* < 0.001) with normal distributed data (Shapiro-Wilk, *P* = 0.883) and equal variances (*P* = 0.273). ^68^Ga-mCXCL12 *I*/*R* was initially 1.4 ± 0.3 at 12 hours after acute MI with a steady and significant increase until day 2 (4.5 ± 0.6; *P* = 0.023). A plateau phase was reached between days 2 and 4, followed by a steady and significant decline up to day 10 after MI (1.3 ± 0.6; *P* = 0.004; see Fig. 5)

### In Vivo Molecular Imaging of Cardiac CXCR4 Expression

**Figure 6:**
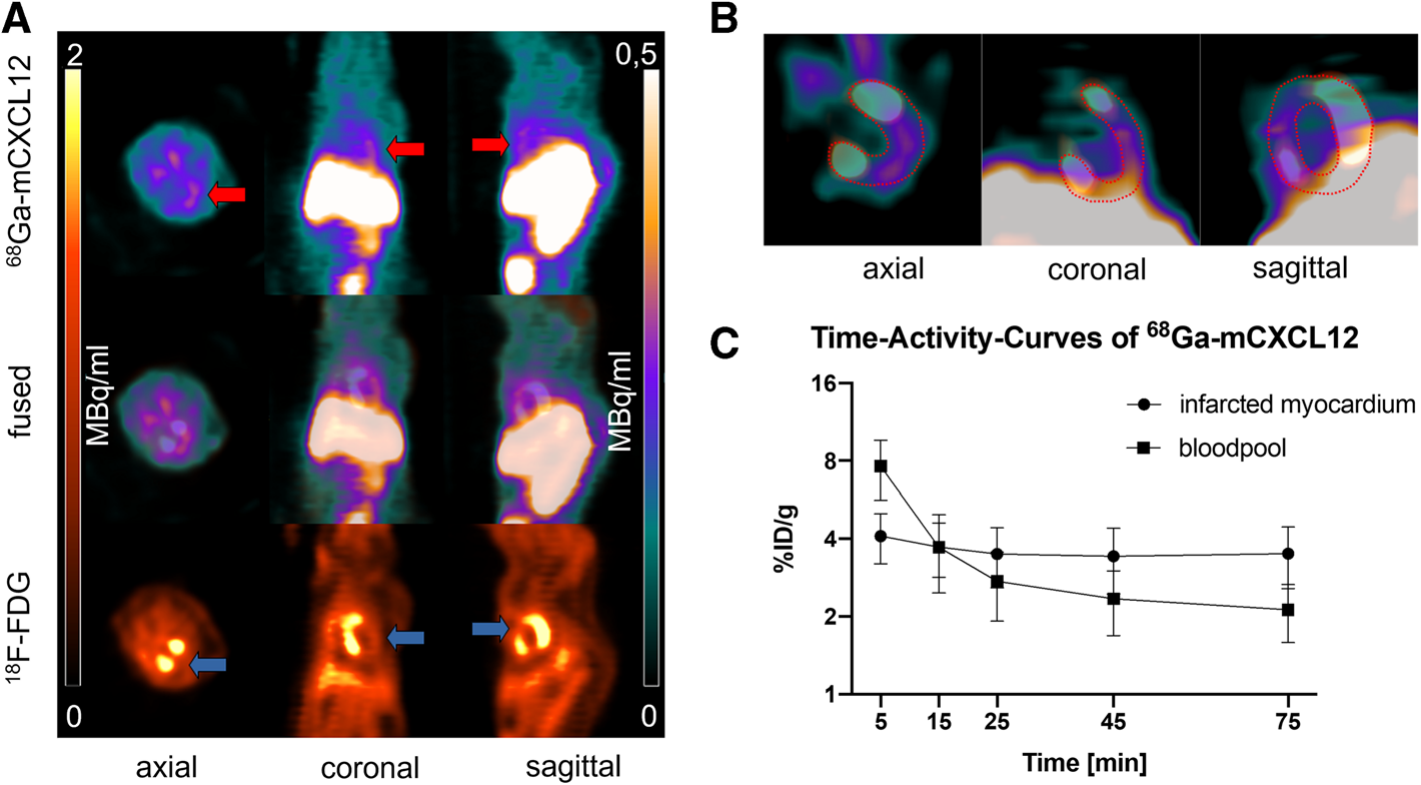
^68^Ga-mCXCL12 PET and time-activity curves. (**A**) PET image on day 3 with increased ^68^Ga-mCXCL12 uptake in the infarcted myocardium (red arrow: ^68^Ga-mCXCL12 uptake; blue arrow: infarcted myocardium). ^18^F-FDG PET showing the viable myocardium. (**B**) Fused and zoomed images of ^68^Ga-mCXCL12 and ^18^F-FDG PET with red dotted lines around the myocardium. (**C**) Representative ^68^Ga-mCXCL12 PET time-activity curves in the myocardium and the blood pool with the best infarct-to-blood pool ratio at the end of the acquisition time. All data represent mean ± SEM

**Figure 7:**
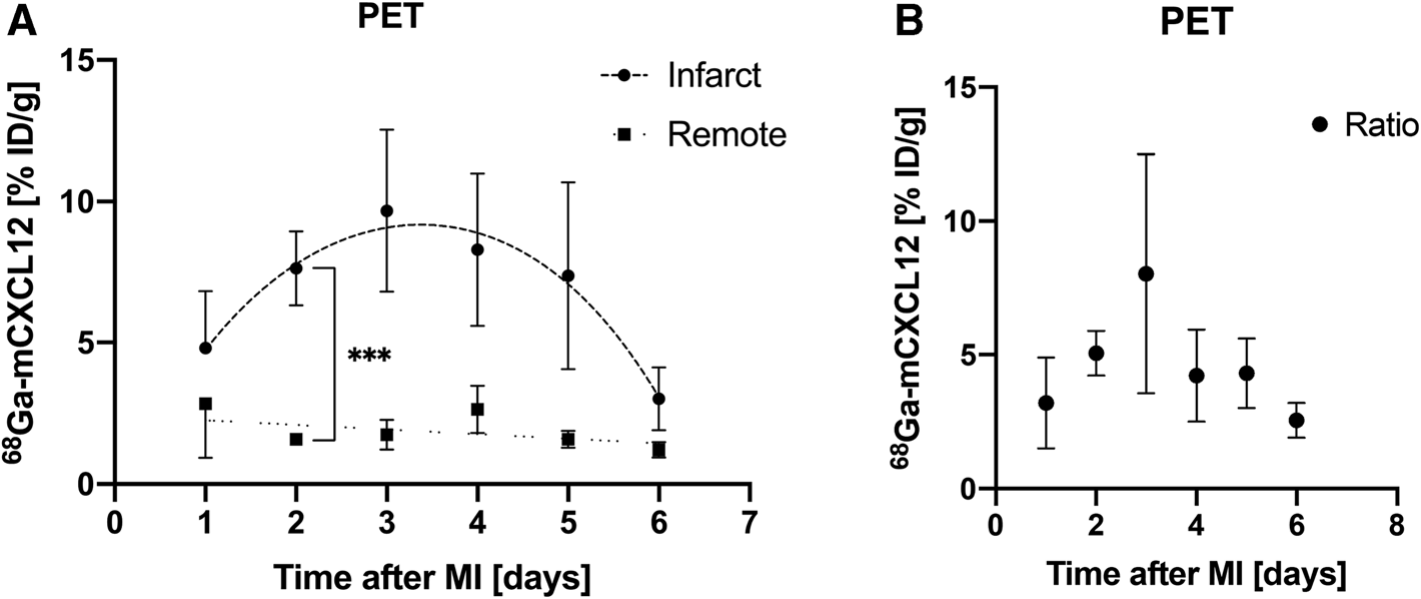
Time-course of CXCR4 expression in PET. (**A**) Percentage injected dose per gram of tissue (%ID/g) in the infarct increasing until day 3 with a consecutive decrease until day 6 (dashed line). No change in ^68^Ga-mCXCL12 PET in the remote myocardium over time (dotted line). For better visualization of the CXCR4 kinetic (%ID/g) over time a fourth-order polynomial regression trendline was calculated, for remote myocardium a linear trendline was plotted. (**B**) Infarct-to-remote ratios (*I*/*R*) of ^68^Ga-mCXCL12 PET over time. All data represent mean ± SEM

Image fusion of ^18^F-FDG and ^68^Ga-mCXCL12 PET scans was performed to allow for proper attribution of CXCR4 uptake to the area of infarction (Fig. 6A; middle row). As visualized in Fig. 7, the PET scan shows an increasing uptake of ^68^Ga-mCXCL12 in the infarcted area. The high uptake in the liver is evident in all three axes of the PET image (Fig. 6A) as already visualized in tracer biodistribution curves (Fig. 3). Figure 6C indicates a constant decrease of the blood pool activity with a constant accumulation in the infarcted area and therefore an optimal infarct-to-remote ratio is obtained between 60 and 90 minutes.

Even though not significant at the 5% level, there was an evidently increasing tracer uptake in the infarcted myocardium from day 1 to day 3 in the PET scan (day 1: 4.6 ± 3.2 %ID/g; day 3: 9.7 ± 5.0 %ID/g), which then reached a plateau phase until day 4 and then again a subsequent decrease until day 6 (2.8 ± 1.0 %ID/g). The tracer uptake in the remote myocardium remained essentially low and stable without any significant changes over time. Day 2 revealed a significantly higher uptake of ^68^Ga-mCXCL12 in the infarcted area as compared to the remote myocardium (see Fig. 7).

### Immunofluorescence Analysis

To confirm that the changes in ^68^Ga-mCXCL12 binding in the heart are associated with the over-expression of CXCR4, we analyzed the CXCR4 expression in infarcted animals by immunofluorescence analysis at day 3 after MI. Evaluation of the infarcted myocardium with an anti-CXCR4 antibody demonstrated visible expression levels of CXCR4 (Fig. A3).

## Discussion

The current study was designed as proof of concept to demonstrate that ex vivo and in vivo molecular imaging of CXCR4 expression in the murine heart using the proposed novel and mouse-specific PET tracer ^68^Ga-mCXCL12 is feasible. Furthermore, our intent was to investigate the CXCR4 expression after initial acute MI over time in a mouse model of permanent LAD occlusion. This might indicate the optimal timeframe for future in vivo imaging studies in preclinical models of ischemic heart disease. Although, due to the lower positron range, which leads to a higher spatial resolution, a ^18^F-based tracer would have been preferable, but considering the kit-like labeling technique, the mouse-specific protein, as well as the widespread availability of generator-produced ^68^Ga, the proposed ^68^Ga-mCXCL12 radioligand is an ideal tracer, potentially available at many research sites.^18^

Our findings reveal an increasing tracer signal in the infarcted area corresponding to an increased cardiac CXCR4 expression peaking between day 3 and 4 as shown by concordant findings in ex vivo autoradiography and PET with a subsequent decrease up to day 10 post MI.

In recent years, ^68^Ga-Pentixafor has been introduced as suitable clinical tracer for imaging CXCR4 expression.^22^ Initially, ^68^Ga-Pentixafor has been used for tumor imaging and in patients with lymphoproliferative disease.^5^ Additionally, in small pilot studies of noninvasive imaging, ^68^Ga-Pentixafor was suitable to visualize myocardial CXCR4 expression in patients after acute MI.^23^-^26^ However, despite high affinity of ^68^Ga-pentixafor for the human CXCR4 receptor, affinity for murine CXCR4 was significantly lower in this study.^5^,^23^ Therefore, the proposed tracer, which uses a murine CXCL12 protein should be more appropriate to visualize murine CXCR4 expression.^18^ The highest mean %ID/g on day 3 for ^68^Ga-Pentixafor reported by Thackeray et al. was 1.5, whereas we observed a mean %ID/g on day 3 of 9.7 indicating the higher specificity of our novel tracer to the murine CXCR4 receptor.^23^ Comparably better results were also observed for the *I*/*R* with an up to threefold higher ratio in our study compared to results obtained with ^68^Ga-pentixafor.

The performed blocking study with a CXCR4 antibody decreased the ^68^Ga-mCXCL12 uptake in the infarcted area to values which were not different from the background level (Fig. 4). This demonstrates a CXCR4-specific binding of ^68^Ga-mCXCL12, suggesting that the ex vivo and in vivo signals obtained using this tracer primarily arises from CXCR4-positive cells. Similar results were reported with the CXCR4-antagonist AMD3100 for ^68^Ga-pentixafor.^23^

By autoradiography and in vivo PET imaging we identified regional CXCR4 upregulation in the infarct region peaking approximately at day 3 post MI, reaching a plateau phase followed by a steady and significant decline which was in good agreement to previously published data.^23^,^27^ Thackeray et al. reported a significantly increased *I*/*R* at day 3 as compared to the control group with a significant decline up to day 7.^23^ Li et al. investigated the CXCR4 expression in a rat ischemia-reperfusion model (*I*/*R*) with ^125^I-pentixather and found a maximum uptake 3 days after *I*/*R*. Signal decreased after 3 days but was still visible 7 days after MI.^27^

However, with our PET imaging approach, the exact source of the cellular signal remains unclear. In fact, the image displays a composition of different cell types, which are present in the respective myocardial region. Based on the observation that our tracer signal peaked at the time point of maximum inflammation in the infarcted animals, points to a high probability that most of the CXCR4 signal is derived from leukocytes.^26^ This is in agreement with previous data, where the ^68^Ga-Pentixafor signal in the infarct area 3 days post MI was associated with increased detection of macrophages and granulocytes by immunostaining.

Zhang et al. demonstrated augmented CXCR4 expression in the infarct zone as early as 24 hours after acute myocardial infarction by immunofluorescent CXCR4 staining in the infarct border zone^13^ with increased levels up to day 7 which corroborates our CXCR4 expression kinetics. Furthermore, flow cytometry analysis detected increased levels of CD45 positive leukocytes in the damaged myocardial region at the same time point.^23^

The regional and systemic inflammatory processes play an important role for left ventricular remodeling after myocardial infarction and the subsequent development of chronic heart failure.^28^ Therefore, CXCR4 expression after MI might be a possible predictor for a beneficial or deleterious post infarct remodeling. A recent study investigated the use of ^68^Ga-Pentixafor in patients after acute MI. The authors were able to demonstrate that imaging of myocardial CXCR4 is feasible up to 2 weeks after acute MI. Furthermore, tracer uptake in the damaged myocardium correlated with smaller scar volumes at follow-up.^25^ Consistently, a single-time treatment with a CXCR4 antagonist resulted in an attenuated ischemia/reperfusion injury.^17^ Interestingly, a continuous blockade of CXCR4 with AMD3100 resulted in impaired survival and reduced cardiac function after acute MI.^15^,^16^ This shows the unmet need to define the exact role of the chemokine receptor CXCR4 in tissue repair and points out that noninvasive imaging of CXCR4 expression after acute MI is of substantial value.

It is well known, that not only the myocardium at risk but also the remote myocardium is affected by the inflammatory process which also provides the basis for adverse left ventricular remodeling.^29^ In our study, the tracer uptake in the remote myocardium remained essentially low and stable over the 6 days. This might be attributed to our rather short imaged period. Gross et al. found a significant decrease in cardiac ^18^F-FDG uptake from day 6 to day 30 after acute MI which might be attributed to an initial elevated inflammatory process in the remote myocardium.^30^ Unfortunately, no data until day 6 was reported by Gross et al. Future studies are therefore warranted focusing on the long-term CXCR4 expression in the remote myocardium as a prognostic marker for cardiac remodeling.

To our knowledge, CXCR4 chemokine imaging has not been translated to other conditions of the heart with increased inflammatory activity so far. Therefore, the murine based ^68^Ga-mCXCL12 should also be used in well-established mouse models of myocarditis^31^ or hypertrophic cardiomyopathy^32^ to further clarify a potential prognostic and therapeutic value of CXCR4 molecular imaging.

### Limitations

Although there was an obvious and increased ^68^Ga-mCXCL12 uptake in the infarcted myocardium as compared to the remote area in autoradiography and PET, only day 2 showed a significantly higher %ID/g (see also Fig. 7). This might be attributed to the partially small group size, which also represents a major limitation of this study. As obvious from the biodistribution data (Fig. 3) and the microPET images (Fig. 6), there is a relatively high blood pool activity as well as a quite-high liver uptake. This represents a major limitation of ^68^Ga-mCXCL12 for cardiac imaging and in particular in small animal models due to spill-over in the myocardial wall. Although the number of introduced chemical modifications is rather low (in average 1.6 per CXCL12 molecule), this represents a non-insignificant fraction of derivatized amino acids because of the small size of the protein. This derivatization might lead to a higher binding potential to proteins in the blood which might lead to a somewhat higher blood pool activity as well as the relatively high liver uptake. A possible strategy to reduce these effects might be to reduce the number of derivatized amino acids although this might on the other hand result in lower achievable molar activities or a site-specific labeling which, however, would preclude the use of endogenous protein. Future studies should include a respiratory movement correction and, if a CT scan is available, an additional spill-over correction. Although we showed that ^68^Ga-mCXCL12 binds mainly to CXCR4, we cannot exclude cross reactivity with other chemokine receptors like ACKR3.

## Conclusion

In summary, molecular imaging with the ^68^Ga-labeled murine protein CXCL12, ^68^Ga-mCXCL12, is feasible to detect CXCR4 upregulation early after acute MI in mice with a peak uptake around day 3. Furthermore, we were able to show the CXCR4-specific uptake of the new radioligand. The development of imaging techniques estimating the immune response in the heart after acute MI could in the future help selecting those patients who are going to benefit from dedicated anti-inflammatory therapies. In addition, it will be the goal of further studies to test a potential prognostic and therapeutic value of CXCR4 molecular imaging and the value of chemokine imaging in more clinically relevant models like myocardial ischemia/reperfusion injury as well as other cardiac inflammatory conditions.

## Electronic supplementary material

Below is the link to the electronic supplementary material.

Supplementary material 1 (DOCX 1758 kb)

Supplementary material 2 (PPTX 495 kb)

Supplementary material 3 (M4A 581 kb)

## Abbreviations

CXCR4: Chemokine receptor 4
CXCL12: C-X-C motif chemokine 12 (synonym: SDF-1)
MI: Myocardial infarction
LAD: Left anterior descending coronary artery
PET: Positron emission tomography
^18^F-FDG: ^18^F-fluorodeoxyglucose
^68^Ga: Gallium-68
%ID/g: Percentage of the injected dose per gram of tissue
*I*/*R*: Infarcted myocardium to remote myocardium ratio

## References

[1] Kircher M, Lapa C (2017). Novel noninvasive nuclear medicine imaging techniques for cardiac inflammation. Curr Cardiovasc Imaging Rep.

[2] Fernández-Ruiz I (2018). Inflammation: New insights from PET imaging. Nat Rev Cardiol.

[3] Lee WW, Marinelli B, van der Laan AM, Sena BF, Gorbatov R, Leuschner F, Dutta P, Iwamoto Y, Ueno T, Begieneman MPV, Niessen HWM, Piek JJ, Vinegoni C, Pittet MJ, Swirski FK, Tawakol A, Di Carli M, Weissleder R, Nahrendorf M (2012). PET/MRI of inflammation in myocardial infarction. J Am Coll Cardiol.

[4] Thackeray JT, Bankstahl JP, Wang Y, Wollert KC, Bengel FM (2015). Clinically relevant strategies for lowering cardiomyocyte glucose uptake for ^18^F-FDG imaging of myocardial inflammation in mice. Eur J Nucl Med Mol Imaging.

[5] Gourni E, Demmer O, Schottelius M, D’Alessandria C, Schulz S, Dijkgraaf I, Schumacher U, Schwaiger M, Kessler H, Wester H-J (2011). PET of CXCR4 expression by a ^68^Ga-labeled highly specific targeted contrast agent. J Nucl Med.

[6] Berger EA (1998). Introduction: HIV co-receptors solve old questions and raise many new ones. Semin Immunol.

[7] Zlotnik A (2006). Chemokines and cancer. Int J Cancer.

[8] Burger JA, Kipps TJ (2006). CXCR4: A key receptor in the crosstalk between tumor cells and their microenvironment. Blood.

[9] Luster AD (1998). Chemokines-chemotactic cytokines that mediate inflammation. N Engl J Med.

[10] Charo IF, Ransohoff RM (2006). The many roles of chemokines and chemokine receptors in inflammation. N Engl J Med.

[11] Gerard C, Rollins BJ (2001). Chemokines and disease. Nat Immunol.

[12] Zaruba M-M, Franz W-M (2010). Role of the SDF-1-CXCR4 axis in stem cell-based therapies for ischemic cardiomyopathy. Expert Opin Biol Ther.

[13] Zhang M, Mal N, Kiedrowski M, Chacko M, Askari AT, Popovic ZB, Koc ON, Penn MS (2007). SDF-1 expression by mesenchymal stem cells results in trophic support of cardiac myocytes after myocardial infarction. FASEB J.

[14] Chen J, Chemaly E, Liang L, Kho C, Lee A, Park J, Altman P, Schecter AD, Hajjar RJ, Tarzami ST (2010). Effects of CXCR4 gene transfer on cardiac function after ischemia-reperfusion injury. Am J Pathol.

[15] Dai S, Yuan F, Mu J, Li C, Chen N, Guo S, Kingery J, Prabhu SD, Bolli R, Rokosh G (2010). Chronic AMD3100 antagonism of SDF-1alpha-CXCR4 exacerbates cardiac dysfunction and remodeling after myocardial infarction. J Mol Cell Cardiol.

[16] Jujo K, Hamada H, Iwakura A, Thorne T, Sekiguchi H, Clarke T, Ito A, Misener S, Tanaka T, Klyachko E, Kobayashi K, Tongers J, Roncalli J, Tsurumi Y, Hagiwara N, Losordo DW (2010). CXCR4 blockade augments bone marrow progenitor cell recruitment to the neovasculature and reduces mortality after myocardial infarction. Proc Natl Acad Sci USA.

[17] Jujo K, Ii M, Sekiguchi H, Klyachko E, Misener S, Tanaka T, Tongers J, Roncalli J, Renault M-A, Thorne T, Ito A, Clarke T, Kamide C, Tsurumi Y, Hagiwara N, Qin G, Asahi M, Losordo DW (2013). CXC-chemokine receptor 4 antagonist AMD3100 promotes cardiac functional recovery after ischemia/reperfusion injury via endothelial nitric oxide synthase-dependent mechanism. Circulation.

[18] Wängler C, Wängler B, Lehner S, Elsner A, Todica A, Bartenstein P, Hacker M, Schirrmacher R (2011). A universally applicable ^68^Ga-labeling technique for proteins. J Nucl Med.

[19] Huber BC, Brunner S, Segeth A, Nathan P, Fischer R, Zaruba MM, Vallaster M, Theiss HD, David R, Gerbitz A, Franz W-M (2011). Parathyroid hormone is a DPP-IV inhibitor and increases SDF-1-driven homing of CXCR4(+) stem cells into the ischaemic heart. Cardiovasc Res.

[20] Rominger A, Brendel M, Burgold S, Keppler K, Baumann K, Xiong G, Mille E, Gildehaus F-J, Carlsen J, Schlichtiger J, Niedermoser S, Wängler B, Cumming P, Steiner H, Herms J, Haass C, Bartenstein P (2013). Longitudinal assessment of cerebral β-amyloid deposition in mice overexpressing Swedish mutant β-amyloid precursor protein using ^18^F-florbetaben PET. J Nucl Med.

[21] Lehner S, Todica A, Brunner S, Uebleis C, Wang H, Wängler C, Herbach N, Herrler T, Böning G, Laubender RP, Cumming P, Schirrmacher R, Franz W, Hacker M (2012). Temporal changes in phosphatidylserine expression and glucose metabolism after myocardial infarction: An in vivo imaging study in mice. Mol Imaging.

[22] Wester HJ, Keller U, Schottelius M, Beer A, Philipp-Abbrederis K, Hoffmann F, Šimeček J, Gerngross C, Lassmann M, Herrmann K, Pellegata N, Rudelius M, Kessler H, Schwaiger M (2015). Disclosing the CXCR4 expression in lymphoproliferative diseases by targeted molecular imaging. Theranostics.

[23] Thackeray JT, Derlin T, Haghikia A, Napp LC, Wang Y, Ross TL, Schäfer A, Tillmanns J, Wester HJ, Wollert KC, Bauersachs J, Bengel FM (2015). Molecular imaging of the chemokine receptor CXCR4 after acute myocardial infarction. JACC Cardiovasc Imaging.

[24] Rischpler C, Nekolla SG, Kossmann H, Dirschinger RJ, Schottelius M, Hyafil F, Wester HJ, Laugwitz KL, Schwaiger M (2016). Upregulated myocardial CXCR4-expression after myocardial infarction assessed by simultaneous GA-68 pentixafor PET/MRI. J Nucl Cardiol.

[25] Reiter T, Kircher M, Schirbel A, Werner RA, Kropf S, Ertl G, Buck AK, Wester H-J, Bauer WR, Lapa C (2018). Imaging of C-X-C motif chemokine receptor CXCR4 expression after myocardial infarction with [^68^Ga]Pentixafor-PET/CT in correlation with cardiac MRI. JACC Cardiovasc Imaging.

[26] Lapa C, Reiter T, Werner RA, Ertl G, Wester H-J, Buck AK, Bauer WR, Herrmann K (2015). [^68^Ga]Pentixafor-PET/CT for imaging of chemokine receptor 4 expression after myocardial infarction. JACC Cardiovasc Imaging.

[27] Li J, Peng C, Guo Z, Shi C, Zhuang R, Hong X, Wang X, Xu D, Zhang P, Zhang D, Liu T, Su X, Zhang X (2018). Radioiodinated pentixather for SPECT imaging of expression of the chemokine receptor CXCR4 in rat myocardial-infarction-reperfusion models. Anal Chem.

[28] Westman PC, Lipinski MJ, Luger D, Waksman R, Bonow RO, Wu E, Epstein SE (2016). Inflammation as a driver of adverse left ventricular remodeling after acute myocardial infarction. J Am Coll Cardiol.

[29] Ruparelia N, Digby JE, Jefferson A, Medway DJ, Neubauer S, Lygate CA, Choudhury RP (2013). Myocardial infarction causes inflammation and leukocyte recruitment at remote sites in the myocardium and in the renal glomerulus. Inflamm Res.

[30] Gross L, Paintmayer L, Lehner S, Brandl L, Brenner C, Grabmaier U, Huber B, Bartenstein P, Theiss H-D, Franz W-M, Massberg S, Todica A, Brunner S (2016). FDG-PET reveals improved cardiac regeneration and attenuated adverse remodelling following Sitagliptin + G-CSF therapy after acute myocardial infarction. Eur Heart J Cardiovasc Imaging.

[31] Brunner S, Todica A, Böning G, Nekolla SG, Wildgruber M, Lehner S, Sauter M, Ubleis C, Klingel K, Cumming P, Franz WM, Hacker M (2012). Left ventricular functional assessment in murine models of ischemic and dilated cardiomyopathy using [^18^ F]FDG-PET: Comparison with cardiac MRI and monitoring erythropoietin therapy. EJNMMI Res.

[32] Todica A, Beetz NL, Günther L, Zacherl MJ, Grabmaier U, Huber B, Bartenstein P, Brunner S, Lehner S (2018). Monitoring of cardiac remodeling in a mouse model of pressure-overload left ventricular hypertrophy with [^18^F]FDG MicroPET. Mol Imaging Biol.

[33] Dealwis C, Fernandez EJ, Thompson DA, Simon RJ, Siani MA, Lolis E (1998). Crystal structure of chemically synthesized [N33A] stromal cell-derived factor 1alpha, a potent ligand for the HIV-1 “fusin” coreceptor. Proc Natl Acad Sci USA.

