# Supplementary material for: Molecular imaging of cardiac CXCR4 expression in a mouse model of acute myocardial infarction using a novel 68Ga-mCXCL12 PET tracer
Source: J Nucl Cardiol. 2020 Jul 16;28(6):2965–75. doi: 10.1007/s12350-020-02262-6 (PMC8709820; doi:10.1007/s12350-020-02262-6)
Supplement: Supplementary file 1 — Supplementary material 1 (DOCX 1758 kb) [file 12350_2020_2262_MOESM1_ESM.docx]

**Supplement:**

On day two after LAD ligation ^68^Ga-mCXCL12 was injected. Hearts (n=4) were extracted at 15, 45, 75 and 105 minutes post injection to determine the optimal time point for the *ex vivo* autoradiography. The hearts were prepared for cutting in the cryomicrotome as described in the manuscript. The slides were exposed to the x-ray image plate and the readout was carried out after 12 hours and the infarct to remote activity ratio (I/R) was calculated.


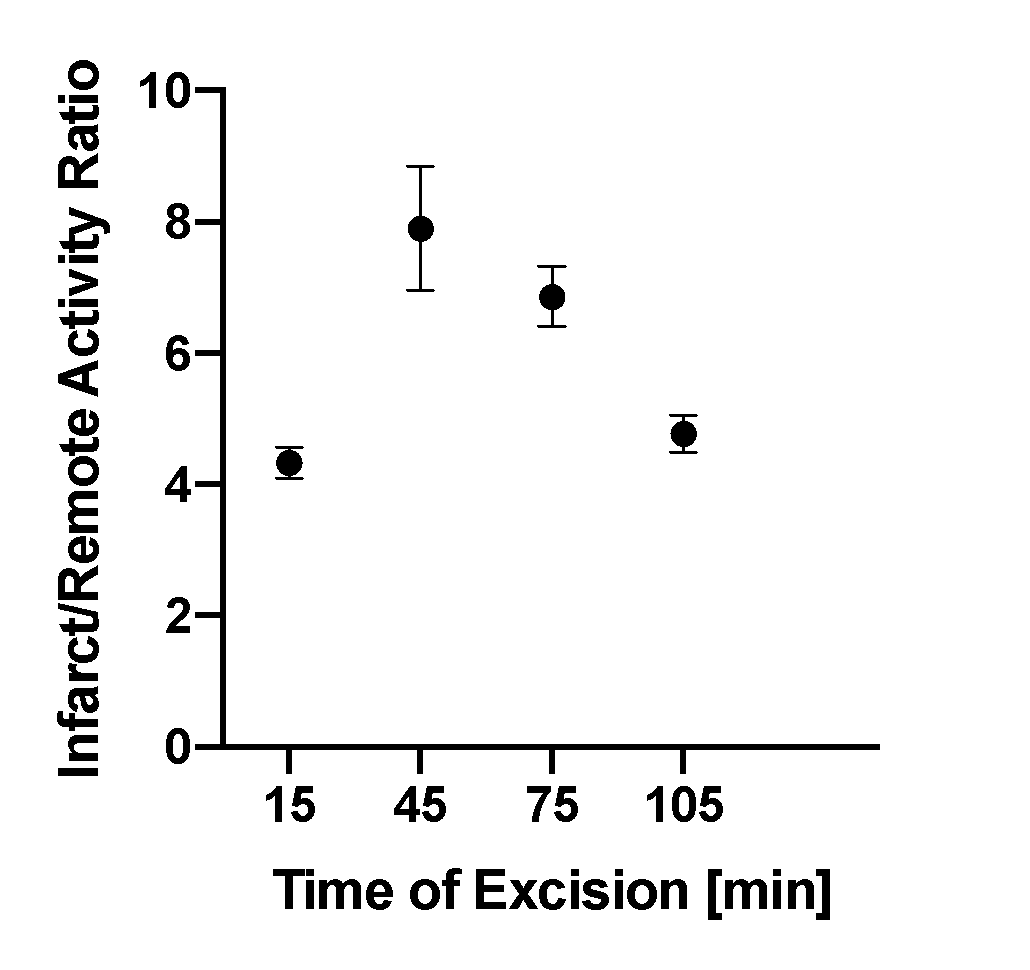


**Figure A1.** Different time points of excision of the heart p.i. ^68^Ga-mCXCL12 with following quantification of infarct to remote activity ratio. All data represent mean ± SEM.

As shown in the figure above there was a fast increase in I/R ratio from 15 min post injection (p.i.) until 45 min p.i. to 7.9±2.7 with a subsequent slow but steady decline until 110 min p.i. Consecutively, all *ex vivo* autoradiography studies were performed at 45 min. p.i.


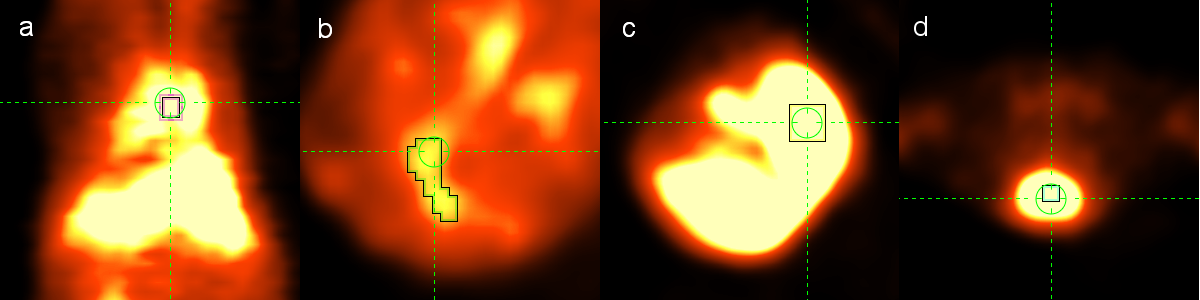


**Figure A2.** Different ROIs in a PET day 3 showing a ^68^Ga-mCXCL12 uptake in: (a) the first 10 min. frame of the blood pool, coronal plane, all other images are in axial plane and from the last frame 60 - 90 min: (b) uptake in the frontal wall of the myocardium (c) liver uptake (d) bladder.


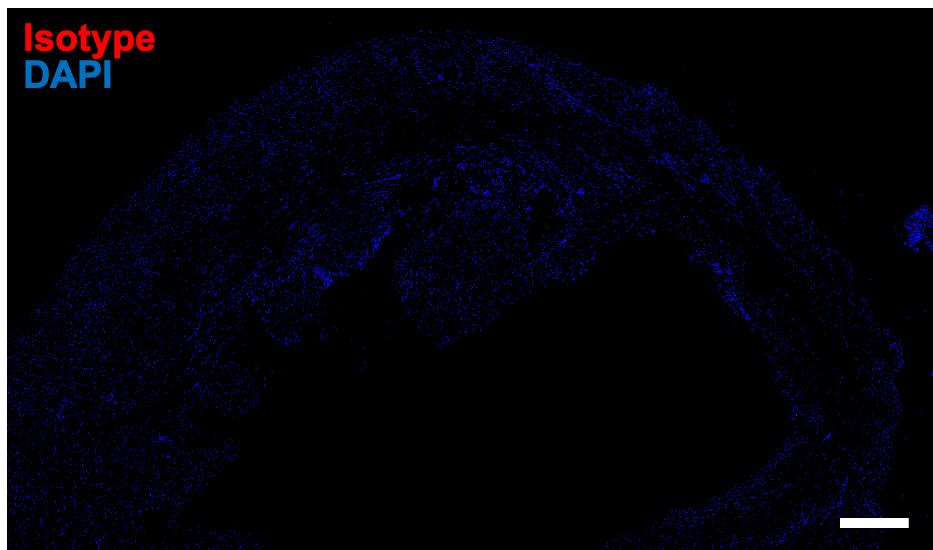


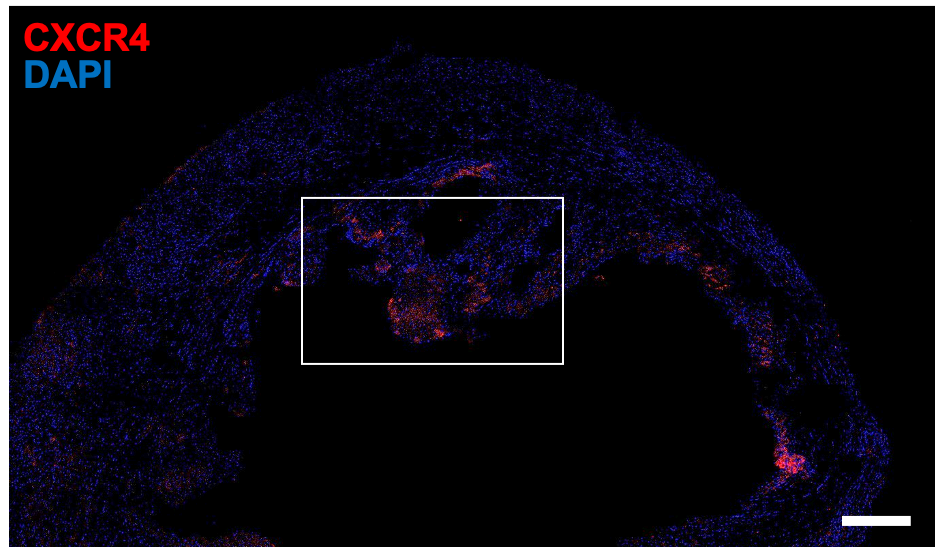


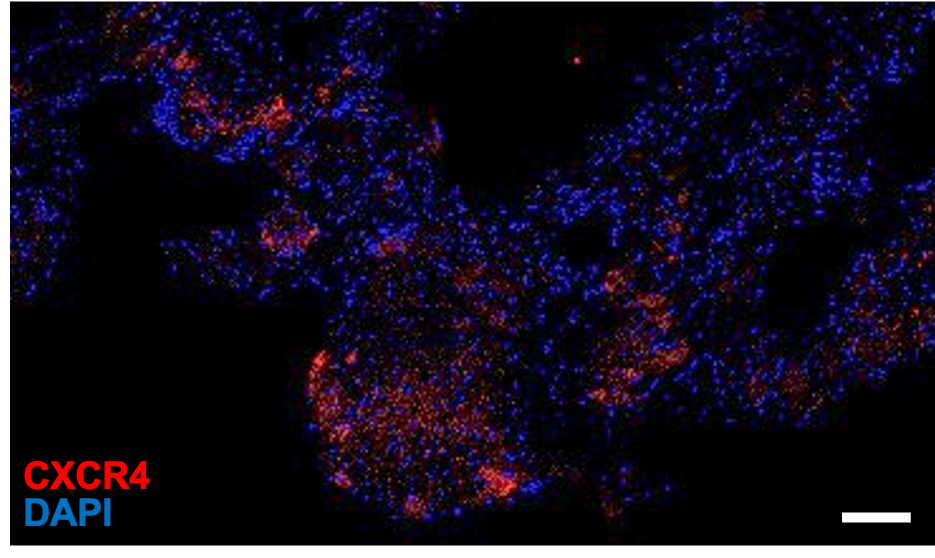


**Figure A3.** CXCR4 Immunofluorescence.

Distribution of CXCR4 showed by immunohistochemistry. Bar 500 µM for lower and 100 µM for higher magnification.
