# Supplementary material for: Molecular imaging of cardiac CXCR4 expression in a mouse model of acute myocardial infarction using a novel 68Ga-mCXCL12 PET tracer
Source: J Nucl Cardiol. 2020 Jul 16;28(6):2965–75. doi: 10.1007/s12350-020-02262-6 (PMC8709820; doi:10.1007/s12350-020-02262-6)
Supplement: Supplementary file 2 — Supplementary material 2 (PPTX 495 kb) [file 12350_2020_2262_MOESM2_ESM.pptx]

## Slide 1
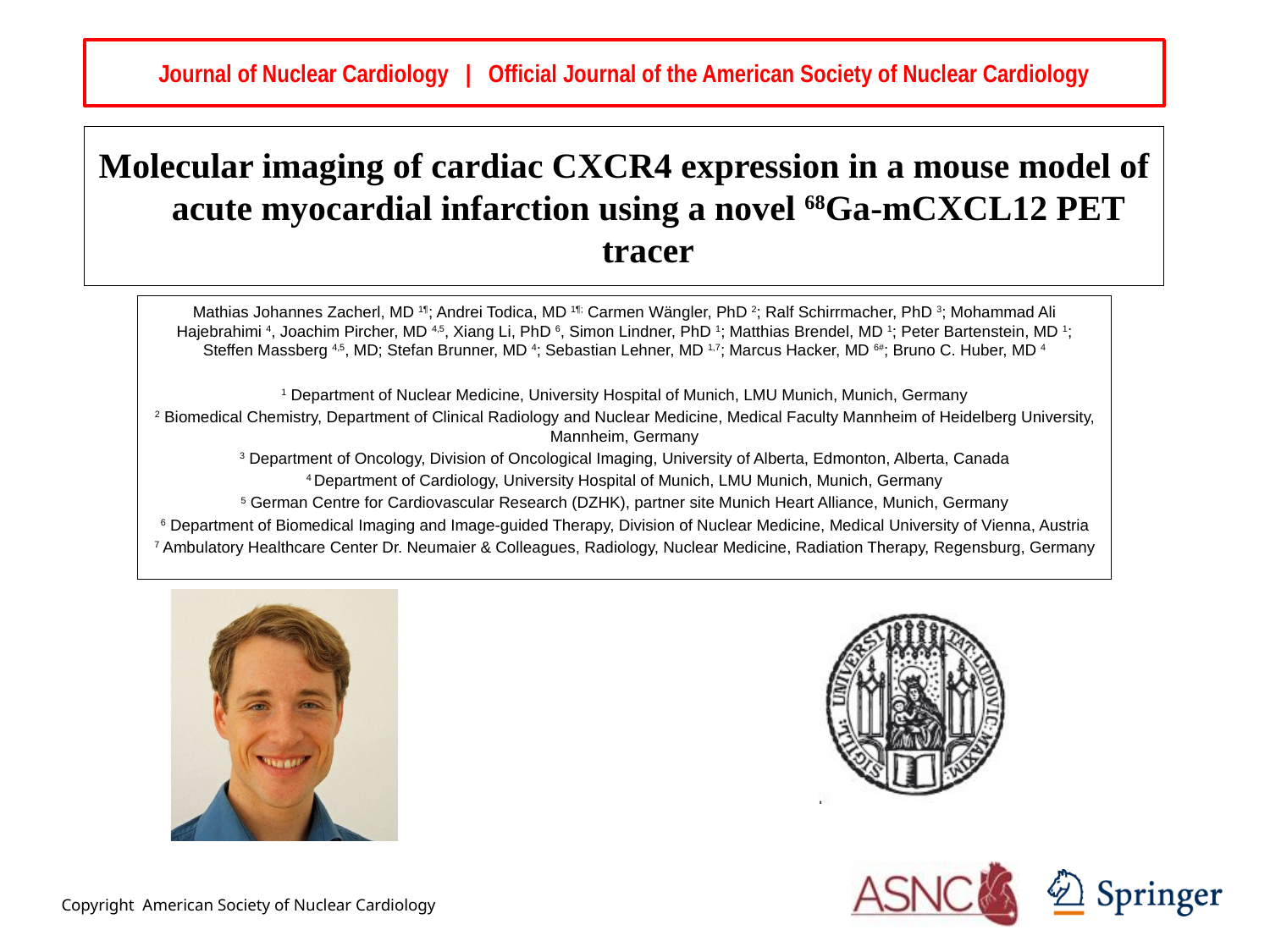

Journal of Nuclear Cardiology | Official Journal of the American Society of Nuclear Cardiology
# Molecular imaging of cardiac CXCR4 expression in a mouse model of acute myocardial infarction using a novel 68Ga-mCXCL12 PET tracer
Mathias Johannes Zacherl, MD 1¶; Andrei Todica, MD 1¶; Carmen Wängler, PhD 2; Ralf Schirrmacher, PhD 3; Mohammad Ali Hajebrahimi 4, Joachim Pircher, MD 4,5, Xiang Li, PhD 6, Simon Lindner, PhD 1; Matthias Brendel, MD 1; Peter Bartenstein, MD 1; Steffen Massberg 4,5, MD; Stefan Brunner, MD 4; Sebastian Lehner, MD 1,7; Marcus Hacker, MD 6#; Bruno C. Huber, MD 4
1 Department of Nuclear Medicine, University Hospital of Munich, LMU Munich, Munich, Germany
2 Biomedical Chemistry, Department of Clinical Radiology and Nuclear Medicine, Medical Faculty Mannheim of Heidelberg University, Mannheim, Germany
3 Department of Oncology, Division of Oncological Imaging, University of Alberta, Edmonton, Alberta, Canada
4 Department of Cardiology, University Hospital of Munich, LMU Munich, Munich, Germany
5 German Centre for Cardiovascular Research (DZHK), partner site Munich Heart Alliance, Munich, Germany
6 Department of Biomedical Imaging and Image-guided Therapy, Division of Nuclear Medicine, Medical University of Vienna, Austria
7 Ambulatory Healthcare Center Dr. Neumaier & Colleagues, Radiology, Nuclear Medicine, Radiation Therapy, Regensburg, Germany
Copyright American Society of Nuclear Cardiology

## Slide 2
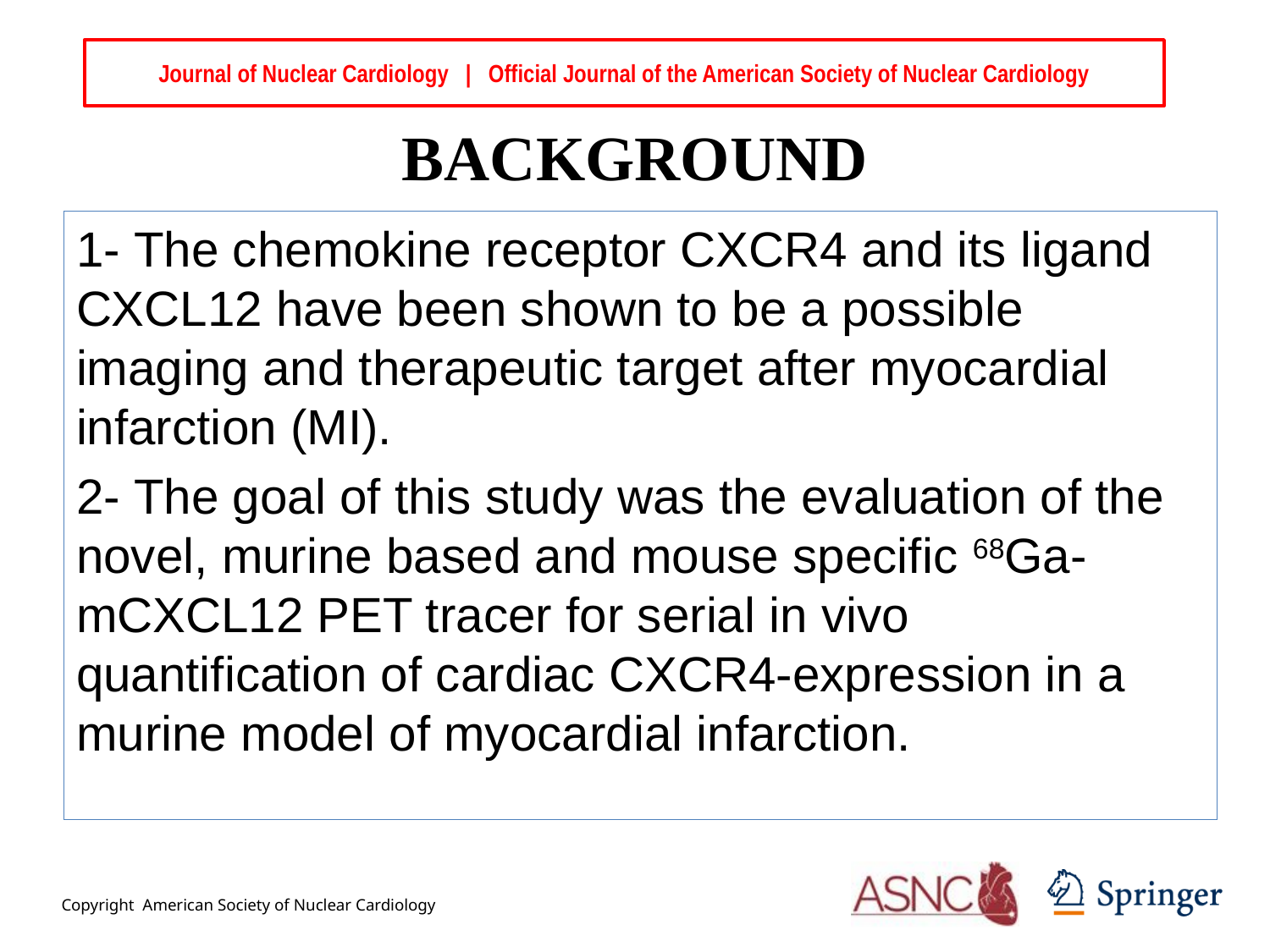

Journal of Nuclear Cardiology | Official Journal of the American Society of Nuclear Cardiology
# BACKGROUND
1- The chemokine receptor CXCR4 and its ligand CXCL12 have been shown to be a possible imaging and therapeutic target after myocardial infarction (MI).
2- The goal of this study was the evaluation of the novel, murine based and mouse specific 68Ga-mCXCL12 PET tracer for serial in vivo quantification of cardiac CXCR4-expression in a murine model of myocardial infarction.
Copyright American Society of Nuclear Cardiology

## Slide 3
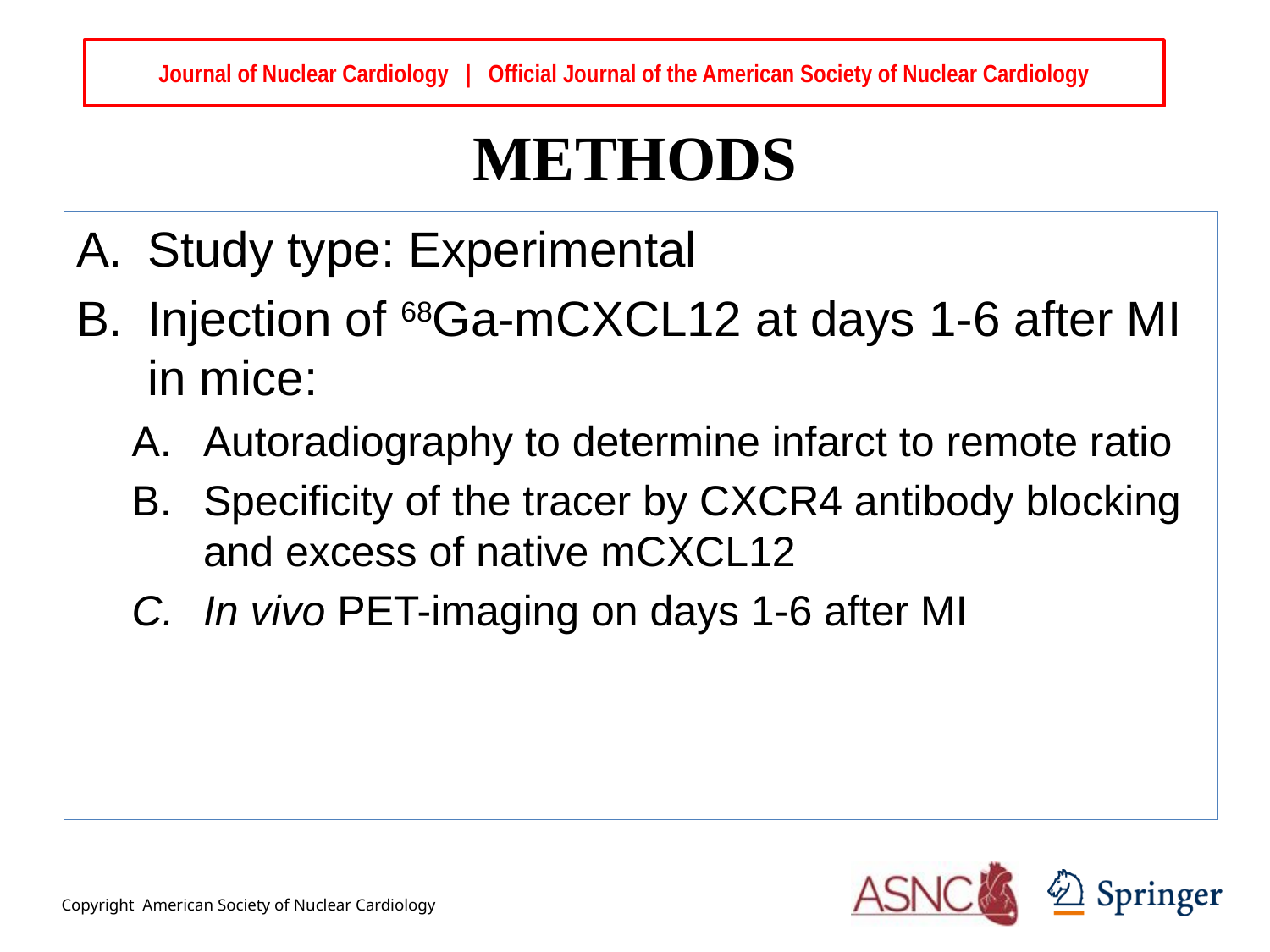

Journal of Nuclear Cardiology | Official Journal of the American Society of Nuclear Cardiology
# METHODS
Study type: Experimental
Injection of 68Ga-mCXCL12 at days 1-6 after MI in mice:
Autoradiography to determine infarct to remote ratio
Specificity of the tracer by CXCR4 antibody blocking and excess of native mCXCL12
In vivo PET-imaging on days 1-6 after MI
Copyright American Society of Nuclear Cardiology

## Slide 4
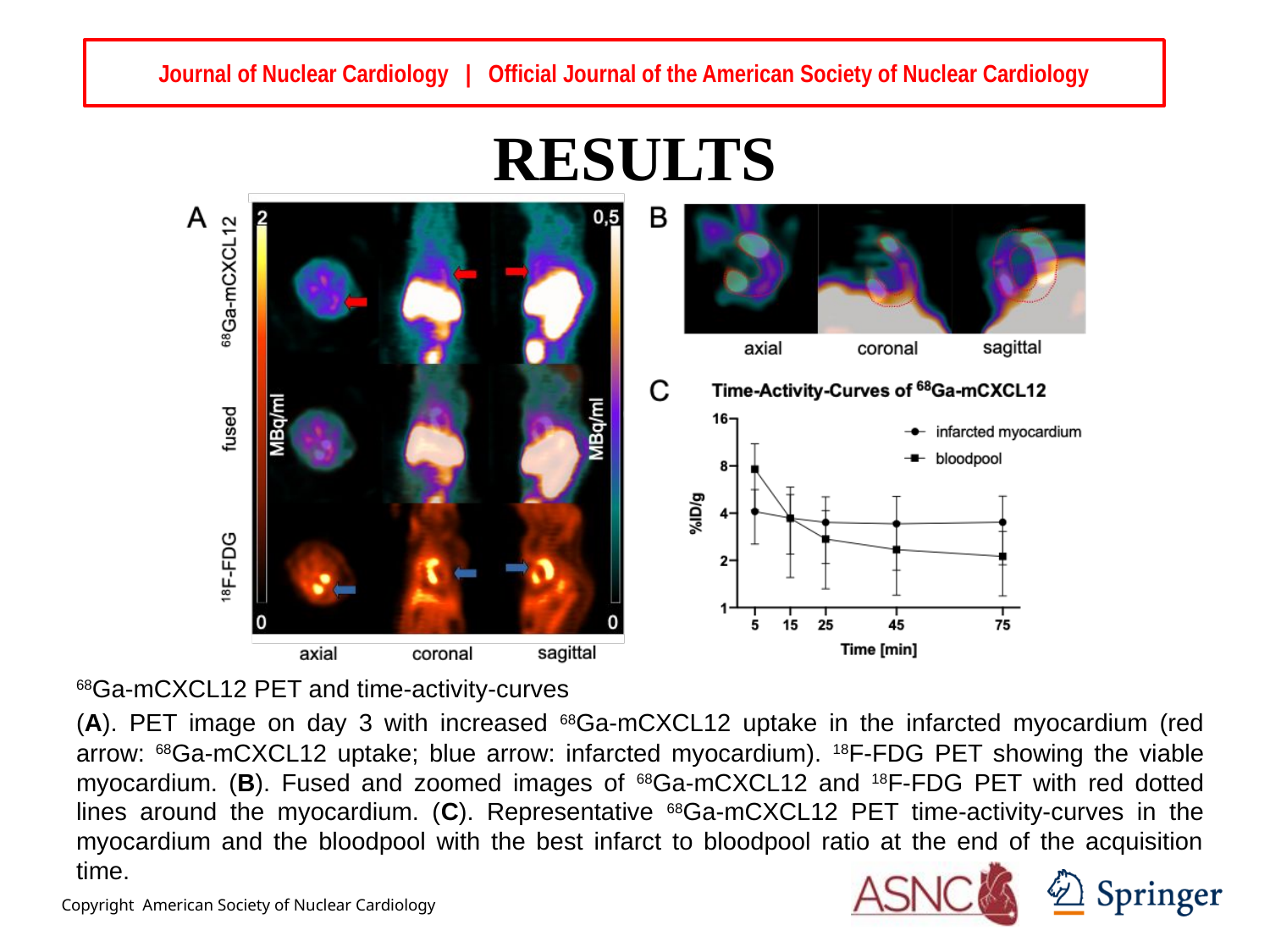

Journal of Nuclear Cardiology | Official Journal of the American Society of Nuclear Cardiology
# RESULTS
68Ga-mCXCL12 PET and time-activity-curves
(A). PET image on day 3 with increased 68Ga-mCXCL12 uptake in the infarcted myocardium (red arrow: 68Ga-mCXCL12 uptake; blue arrow: infarcted myocardium). 18F-FDG PET showing the viable myocardium. (B). Fused and zoomed images of 68Ga-mCXCL12 and 18F-FDG PET with red dotted lines around the myocardium. (C). Representative 68Ga-mCXCL12 PET time-activity-curves in the myocardium and the bloodpool with the best infarct to bloodpool ratio at the end of the acquisition time.
Copyright American Society of Nuclear Cardiology

## Slide 5
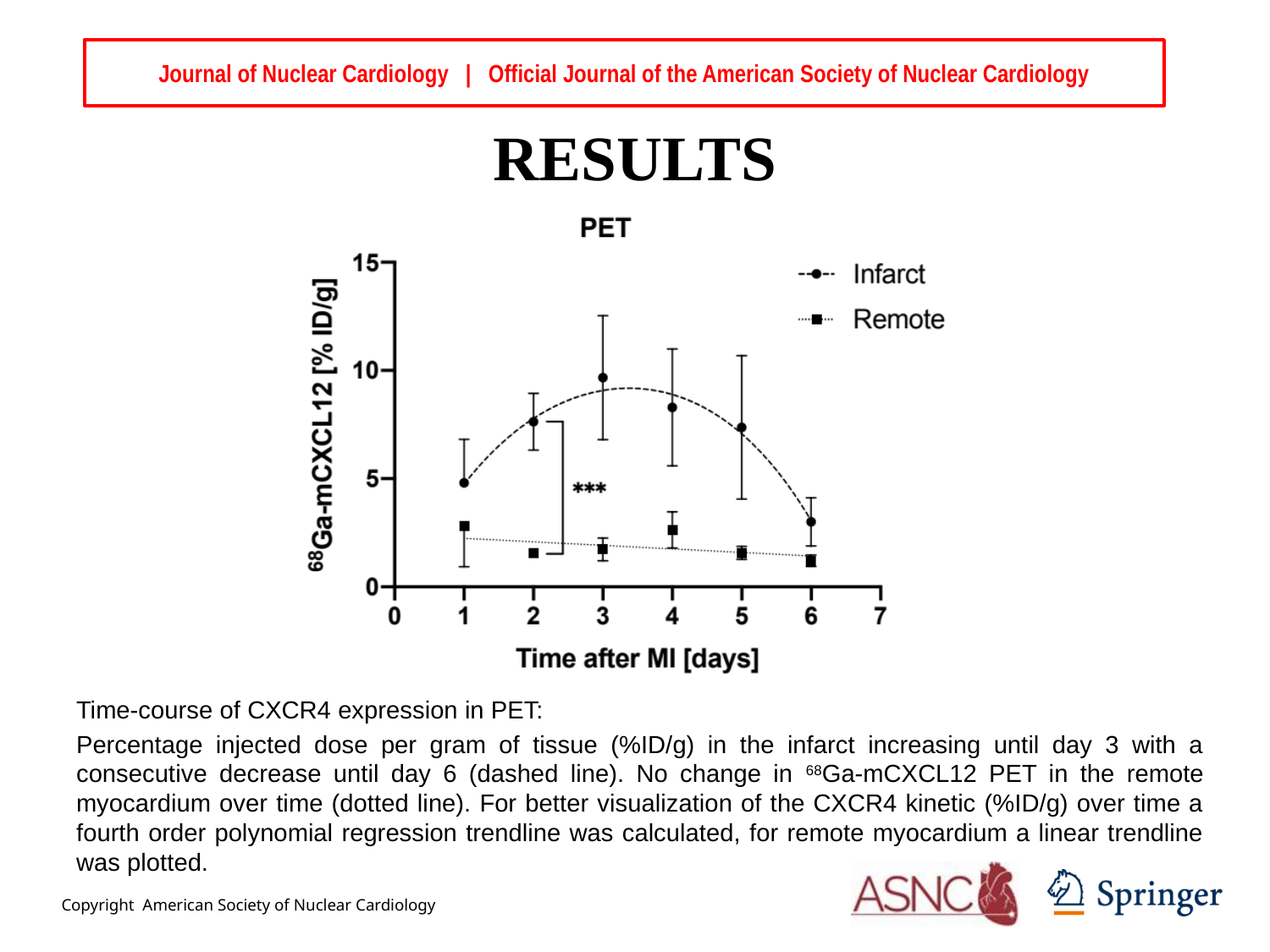

Journal of Nuclear Cardiology | Official Journal of the American Society of Nuclear Cardiology
# RESULTS
Time-course of CXCR4 expression in PET:
Percentage injected dose per gram of tissue (%ID/g) in the infarct increasing until day 3 with a consecutive decrease until day 6 (dashed line). No change in 68Ga-mCXCL12 PET in the remote myocardium over time (dotted line). For better visualization of the CXCR4 kinetic (%ID/g) over time a fourth order polynomial regression trendline was calculated, for remote myocardium a linear trendline was plotted.
Copyright American Society of Nuclear Cardiology

## Slide 6
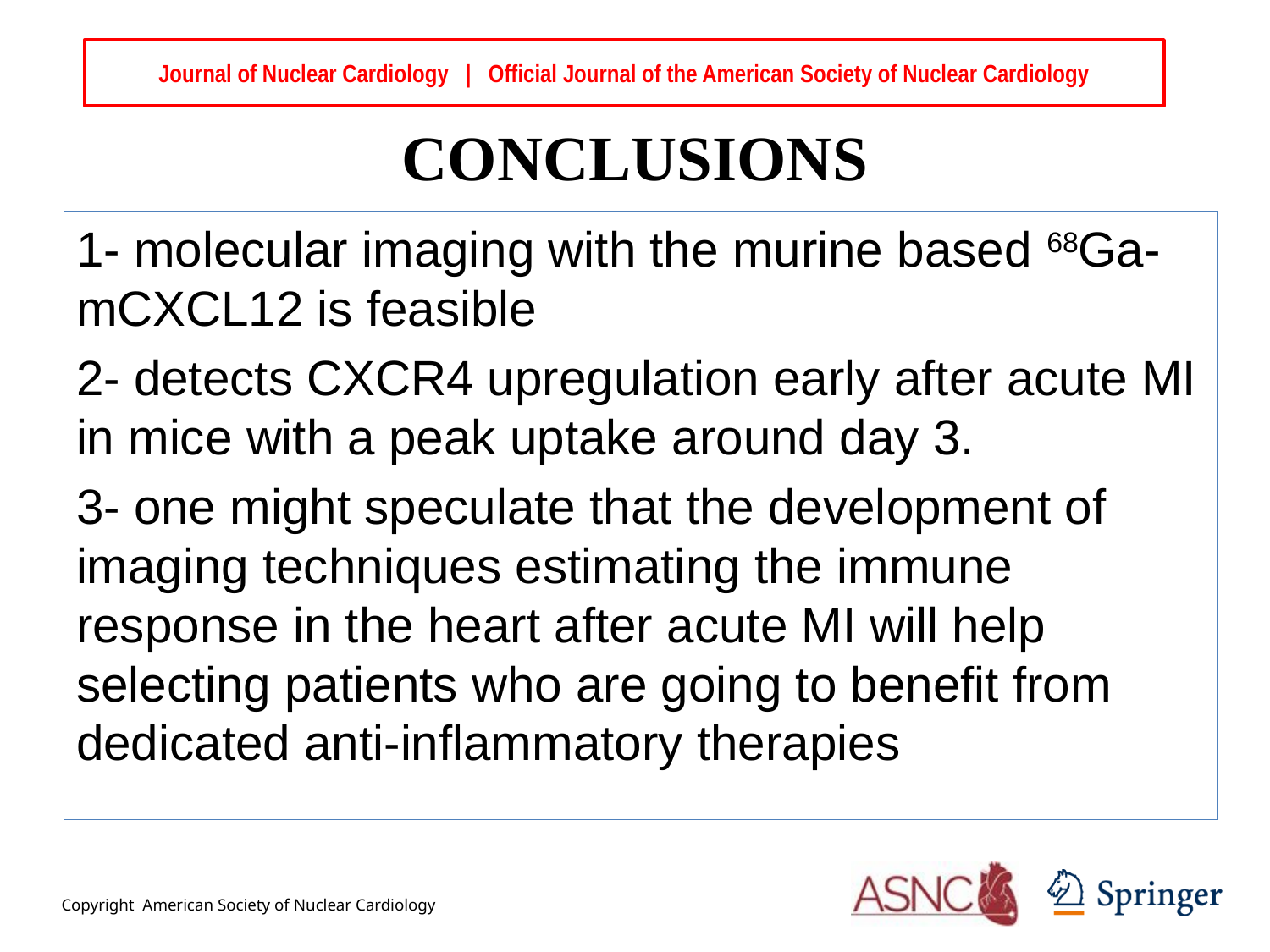

Journal of Nuclear Cardiology | Official Journal of the American Society of Nuclear Cardiology
# CONCLUSIONS
1- molecular imaging with the murine based 68Ga-mCXCL12 is feasible
2- detects CXCR4 upregulation early after acute MI in mice with a peak uptake around day 3.
3- one might speculate that the development of imaging techniques estimating the immune response in the heart after acute MI will help selecting patients who are going to benefit from dedicated anti-inflammatory therapies
Copyright American Society of Nuclear Cardiology
